# Relationships between Jumping Performance and Psychological Readiness to Return to Sport 6 Months Following Anterior Cruciate Ligament Reconstruction: A Cross-Sectional Study

**DOI:** 10.3390/jcm12020626

**Published:** 2023-01-12

**Authors:** Claudio Legnani, Matteo Del Re, Marco Viganò, Giuseppe M. Peretti, Enrico Borgo, Alberto Ventura

**Affiliations:** 1 Sport Traumatology and Minimally Invasive Surgery Center, IRCCS Istituto Ortopedico Galeazzi, 20161 Milan, Italy; 2 Department of Orthopedics and Traumatology, University of Milan, 20133 Milan, Italy; 3 IRCCS Istituto Ortopedico Galeazzi, 20161 Milan, Italy; 4 Department of Biomedical Sciences for Health, University of Milan, 20133 Milan, Italy

**Keywords:** anterior cruciate ligament, ACL reconstruction, return to sports, ACL-RSI, psychological readiness, vertical jump

## Abstract

Background: Investigating the relationship between functional capacity and psychological readiness is of paramount importance when planning sport resumption following knee surgery. The aim of this study was to prospectively assess clinical and functional outcomes in athletes 6 months after primary anterior cruciate ligament (ACL) reconstruction and to evaluate whether jumping ability is related to psychological readiness to return to sport following ACL surgery. Methods: Patients who underwent ACL reconstruction were prospectively enrolled and evaluated pre-operatively and 6 months after surgery. Assessment included Lysholm score, International Knee Documentation Committee (IKDC) Subjective Knee Form, Tegner activity level, and the ACL–Return to Sport after Injury (ACL-RSI) scale. Jumping ability was instrumentally assessed by an infrared optical acquisition system using a test battery including mono- and bipodalic vertical jump and a side hop test. Patients were dichotomized by ACL-RSI into two groups: group A (ACL-RSI > 60), and group B (ACL-RSI < 60). Results: Overall, 29 males and two females from the original study group of 37 patients (84%) were available for clinical evaluation. Mean age at surgery was 34.2 years (SD 11.3). Mean body mass index (BMI) was 25.4 (SD 3.7). Mean overall Lysholm, IKDC, and ACL-RSI scores increased from pre-operatively (*p* < 0.001). No differences in Tegner score were reported (*p* = 0.161). Similarly, improvement in most variables regarding jumping ability were observed at follow-up (*p* < 0.05). According to ACL-RSI, 20 subjects were allocated in group A (ACL-RSI > 60), while 11 were allocated in group B (ACL-RSI < 60). A statistically significant difference in favor of patients in group A was recorded for the post-operative Lysholm and Tegner score, as well as Side Hop test LSI level (*p* < 0.05), while a trend for IKDC was observed without statistical significance (*p* = 0.065). Conclusions: Patients with higher values of ACL-RSI scores showed better functional and clinical outcomes as well as improved performance 6 months after ACL reconstruction

## 1. Introduction

The treatment of anterior cruciate ligament (ACL) rupture in the physically active population is often surgical. Patients treated with ACL reconstruction usually aim to return to sport activities they practiced before injury [1,2,3].

However, a significant number of patients ranging between 37% and 56% are not able to return to sport at pre-injury level [4]. Reasons for that include the persistence of knee strength and neuromuscular deficits, kinesiophobia, and fear of re-injury [5]. Increased risks of re-rupture have been reported within the first two years after surgery, especially in the youngest patients [6,7]. For these reasons, in an attempt to reduce risk factors and to determine the appropriate timing for resuming sports activities, test batteries have been developed. These include but are not limited to jumping abilities assessment, strength tests, and psychological readiness scoring systems [8,9,10,11]. Tests measuring muscular power and reactivity through vertical jump are demonstrated to be reliable instruments and useful predictors for the return to sports [11,12]. Horizontal hop tests have been widely adopted to detect functional asymmetries between limbs [13,14], although their usefulness has been questioned as they are demonstrated to overestimate the involved limb’s function following ACL surgery [15,16]. Investigating the relationship between functional capacity and psychological readiness is of paramount importance when planning sport resumption following ACL reconstruction [9]. Therefore, vertical jump tests have been advocated to better analyze knee biomechanics, thus identifying lower limb asymmetries more accurately, and give a more reliable prediction on the return-to-sport ability [11].

The ACL–Return to Sport after Injury scale (ACL-RSI) is considered a reliable tool to assess psychological readiness to return to sport after ACL surgery [17,18]. It has been demonstrated that patients with lower ACL-RSI scores are at higher risk for ACL re-rupture [19], and recently much emphasis is given to the recovery of psychological responses as a criterion used to clear athletes to return to sport following ACL reconstruction.

The aim of this study was to prospectively assess outcomes of athletes who had undergone primary ACL reconstruction at 6 months after surgery, and to evaluate whether psychological readiness affects functional and clinical results as well as jumping ability. The hypothesis was that athletes’ psychological readiness is strictly related to jumping performance.

## 2. Materials and Methods

### 2.1. Patient Recruitment

From January 2021 to the end of December 2021, 37 consecutive patients who underwent ACL reconstruction at the Department of Sport Traumatology and Minimally Invasive Surgery of our Institution were prospectively enrolled and evaluated pre-operatively and 6 months after surgery. Diagnosis was done with clinical examination and knee magnetic resonance imaging, and further verified arthroscopically. All surgeries were performed by the same senior surgeon.

Inclusion criteria were: primary unilateral ACL reconstruction; age ≥ 18 years and ≤45 years at surgery; time from injury to surgery ≥ 2 weeks and ≤12 months; participation in sporting activity; same postoperative rehabilitation protocol. Exclusion criteria were: past history of ligamental surgery on the same or contralateral knee; concomitant surgical procedures with the exception of treatment for meniscal pathology; pregnancy; inability to complete clinical and functional testing.

The study received Institutional Review Board approval (IRB number: 57/INT/2020, released from IRCCS San Raffaele Hospital, Milan, Italy). All participants signed informed consent.

### 2.2. Surgical Technique and Rehabilitation Protocol

All patients underwent arthroscopic-assisted ACL reconstruction using doubled autologous hamstring graft [6]. Tibial tunnel was drilled using a 55° guide (Acufex; Smith & Nephew, Andover, MA, USA) using as reference the posterior cruciate ligament, while the femoral half-tunnel was prepared either through the medial portal or with a trans-tibial technique. Fixation was achieved proximally with a cortical suspension device (Retrobutton; Arthrex Inc., Naples, FL, USA) and distally through a bioadsorbable interference screw (Milagro; DePuy Mitek, Raynham, MA, USA). A brace-free rehabilitation protocol starting the day after surgery was adopted in all patients, with immediate regaining of extension, isometric exercises, and walking with crutches with partial weight bearing for the first 3 weeks. Swimming and indoor cycling were allowed after 12 weeks, while after 5 months a protocol of jump technique training and plyometric exercises was started.

### 2.3. Follow-Up Assessment

Assessment included Lysholm score, International Knee Documentation Committee (IKDC) Subjective Knee Form, Tegner activity level, and ACL-RSI scale. The Lysholm score is a 100-point maximum subjective questionnaire developed to evaluate knee functional status after ACL surgery [20]. The IKDC subjective score is a clinical questionnaire that assesses the functional status of the knee ranging from 0 to 100, with 100 indicating no limitations [21]. The Tegner activity scale is designed to grade patient’s activity level based on work and sports activities from 0 (lowest level) to 10 [22]. ACL-RSI is a previously validated 12-item questionnaire that allows evaluating psychological readiness to return to sport following ACL injury or surgery on a scale from 0 to 100. The Italian version of the ACL-RSI used in the present study showed excellent internal consistency and reliability [18].

Jumping ability was assessed using a test battery modified from Gustavsson et al. [23], using the OptoGait (Microgate, Bolzano, Italy) system. This device allows us to calculate jumping height (in centimeters) from flight time (in milliseconds) measured by an infrared optical acquisition system connected to a software (OptoGait analysis software, version 3.22; Microgate).

**Figure 1:**
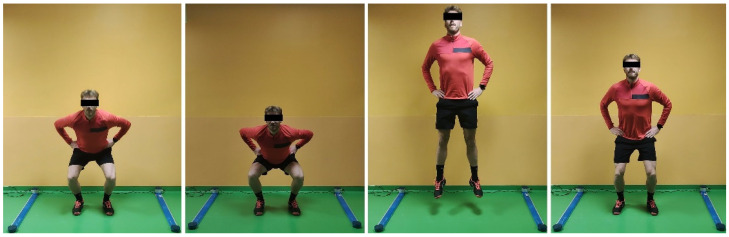
Bipodalic squat jump.

**Figure 2:**
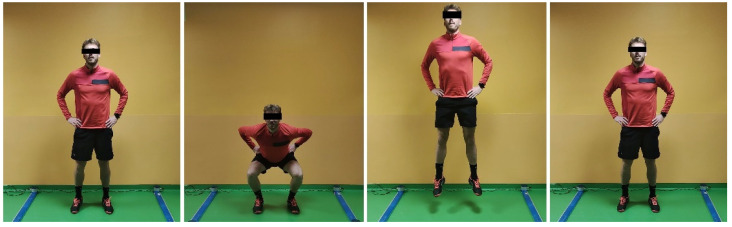
Bipodalic countermovement jump.

**Figure 3:**
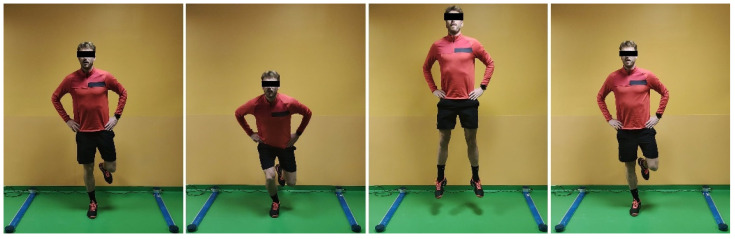
Monopodalic countermovement jump.

**Figure 4:**
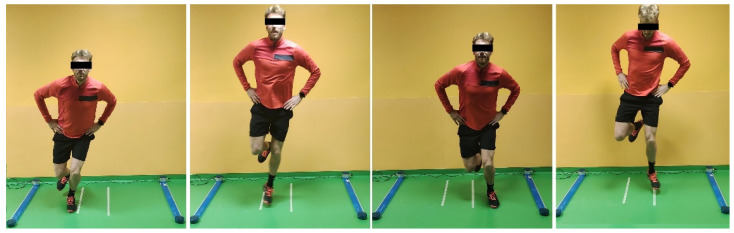
Monopodalic side hop test.

Two types of bipodalic jumps were tested: squat jump (SJ), and countermovement jump (CMJ) (Figure 1 and Figure 2). Then, monopodalic jumps were performed: CMJ, and side hop test (Figure 3 and Figure 4). Each of them was performed with the uninjured limb first, followed by the injured. The SJ consisted of a vertical jump performed from a half-squat position with the knees flexed at 90°. The CMJ was performed in the same fashion, with the exception that the starting position was an upright position, and the jump was performed after a quick sinking. Every jump was performed 3 times, and the average value was recorded. The side hop test consisted of performing as many jumps as possible during a period of 30 s between two parallel strips on the floor put at a distance of 30 cm without touching the strips (a maximum of 25% error was allowed; otherwise, the test was repeated). Limb Symmetry Index (LSI) was calculated as a percentage of test performance on the injured/operated limb compared to the contralateral limb.

### 2.4. Statistical Analysis

Statistical analyses were performed using Graphpad Prism v8.0 (Prism software, La Jolla, San Diego, CA, USA). Shapiro–Wilk test was used to test the distribution of each variable. In case of normal distribution, paired Student t tests were used to assess differences between pre-operative and 6-month values. Patients were dichotomized by post-operative ACL-RSI score into two groups: group A (ACL-RSI > 60), and group B (ACL-RSI < 60); unpaired t test were used to evaluate differences between the two groups. In case of non-normal distribution, the same assessments were performed by Wilcoxon signed-rank test for matched pairs and Mann–Whitney U test, respectively. Difference between proportions were assessed by Fisher’s exact test. *p* values < 0.05 were considered statistically significant. Post-hoc power analysis was performed for the test evaluating the improvements in Lysholm, ACL-RSI, and IKDC after surgery. For a test with alpha = 0.05, the study sample size provided a test power > 90% for each score.

## 3. Results

### 3.1. Demographic Data

Six patients (16%) were lost at follow-up. Overall, there were 29 males and two females available for clinical evaluation. Mean age at surgery was 34.2 years (SD 11.3). Mean body mass index (BMI) was 25.4 (SD 3.7). Average time interval between injury to surgery was 2.7 months (SD: 1.1). Patients’ demographics and anthropometric data are reported in Table 1. At the time of injury, 21 patients practiced contact sports (soccer, basketball, rugby). Noncontact sports (volleyball, skiing, cycling, running, swimming, tennis) were practiced in 28 cases. Eleven patients practiced sport at an agonistic level, 20 were amateurs.

### 3.2. Subjective Knee Function

**Figure 5:**
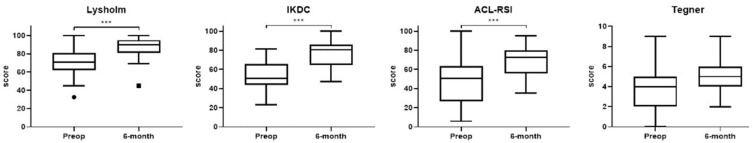
Box-plots showing differences in outcome scores from pre-operative to 6-month follow-up after surgery. The black line inside the box represents median value. The lowest bar represents the minimum value, the bottom and top of the boxes represent the interquartile range (25th and 75th percentiles), and the top bar represents the maximum value. Points outside the limits represent outliers *** *p* < 0.001.

The mean overall Lysholm score increased from a pre-operative mean of 68.4 (SD: 15.6) to 87.1 (SD: 11.2), showing a statistically significant difference (*p* < 0.001). IKDC subjective score improved from 51.9 (SD: 13.0) to 77.1 (SD: 14.6) (*p* < 0.001). Similarly, ACL-RSI changed from 46.2 (SD: 23.2) to 68.3 (SD: 16.2) (*p* < 0.001). Concerning Tegner activity level, no statistically significant differences were reported between pre- and post-operative status (mean value 4.2, SD: 2.4, and 5.1, SD: 1.8, respectively, *p* = 0.161) (Table 2, Figure 5).

### 3.3. Jump Battery Tests

**Figure 6:**
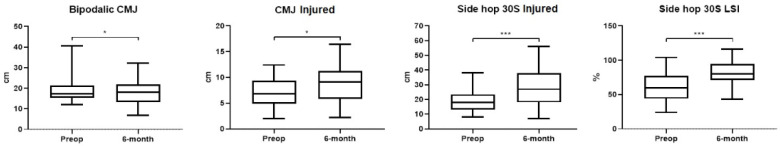
Box-plots showing differences in jumping performances from pre-operative to 6-month follow-up after surgery. The black line inside the box represents median value. The lowest bar represents the minimum value, the bottom and top of the boxes represent the interquartile range (25th and 75th percentiles), and the top bar represents the maximum value. * *p* < 0.05; *** *p* < 0.001.

The following variables significantly improved at follow-up compared to pre-operatory status: bipodalic CMJ (*p* = 0.049), monopodalic CMJ on the injured limb (*p* = 0.037), CMJ LSI (*p* = 0.037), 30 s Side Hop test on the injured limb (*p* < 0.001), and Side Hop test LSI (*p* < 0.001) (Figure 6). No differences in other jump tests were recorded (*p* = n.s.).

### 3.4. Results According to ACL-RSI

Patients were dichotomized by post-operative ACL-RSI into two groups: 20 subjects were allocated in group A (ACL-RSI > 60), while 11 were allocated in group B (ACL-RSI < 60). No statistically significant differences between these two groups were reported concerning average Lysholm, IKDC, ACL-RSI, and Tegner score (*p* = 0.92, 0.69, 0.21, and 0.44, respectively) at baseline.

**Figure 7:**
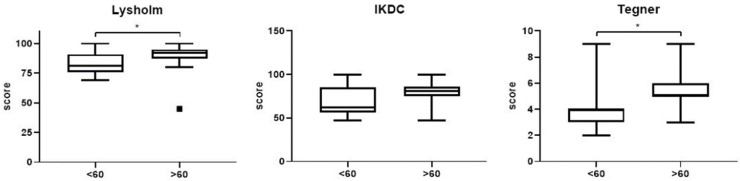
Box-plots showing differences between the two groups with the same change compared to the baseline for the various parameters. The black line inside the box represents median value. The lowest bar represents the 10th percentile, the bottom and top of the boxes represent the interquartile range (25th and 75th percentiles), and the top bar represents the 90th percentile. * *p* < 0.05.

**Figure 8:**
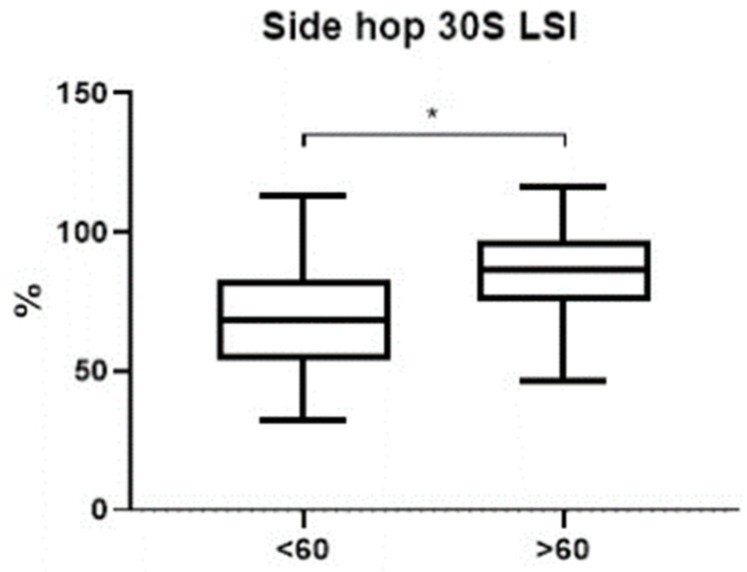
Box-plot showing differences in Side Hop test LSI between the two groups. The black line inside the box represents median value. The lowest bar represents the minimum value, the bottom and top of the box represent the interquartile range (25th and 75th percentiles), and the top bar represents the maximum value. * *p* < 0.05.

Between groups, comparisons were performed using individual changes with respect to baseline, in order to adjust for possible biases. A statistically significant difference in favour of group A was recorded for Lysholm score (*p* = 0.020) and Tegner activity level (*p* = 0.006), while a trend for IKDC was observed without statistical significance (*p* = 0.065) (Figure 7). Considering jump tests, the following variable was significantly higher in group A: Side Hop test LSI (*p* < 0.05) (Figure 8). No differences concerning other jump tests were recorded between the two groups (*p* = n.s.).

## 4. Discussion

The most important finding of the present study was that patients with higher values of ACL-RSI scores showed better functional clinical outcomes according to Lysholm and Tegner scores as well as improved performance as measured with side-hop test six months after ACL reconstruction.

According to our results, six months after surgery, subjective knee function according to point-scales significantly improved compared to pre-operatory status, thus supporting the efficacy of hamstring ACL reconstruction in athletes.

Functional tests are commonly used to assess the return-to-sport ability following ACL reconstruction. In our cohort of patients, a test battery of vertical jumps measured with an optical acquisition equipment was adopted to evaluate jump performance following ACL surgery. Assessment of vertical jump to detect function deficits of the lower limb with ACL injury or following ACL surgery has been previously reported [11,12,24]. The battery of vertical jump tests used in the present investigation involved bipodalic SJ, bipodalic and monopodalic CMJs, and 30 s side hop test. Such tests, by measuring jump height as an expression of knee explosiveness, coordination, and dynamic knee stability, are reliable predictors for the return to sport after ACL surgery. Based on the results of these test batteries, LSI was calculated to evaluate knee status and patient ability to return to cutting and pivoting sports.

Six months after surgery, the ability to perform bipodalic CMJ, monopodalic CMJ on the injured limb, and a 30 s Side Hop test on the injured limb significantly improved at follow-up compared to pre-operatory status. Similarly, an improvement in LSI recorded while performing CMJ and Side Hop test LSI was observed.

Previous studies investigated the relationship between psychological readiness and functional performance [13,25,26]. The ACL-RSI score demonstrated to be a reliable scoring scale while evaluating psychological readiness for return to sports after ACL reconstruction [17,18]. In the study by McPherson et al., patients who underwent ACL re-rupture trended toward lower ACL-RSI scores [19], while according to Sadeqi et al., ACL-RSI score ≥ 60 months after surgery is a reliable predictor of return to preinjury sport [27]. According to the study by Aizawa et al., in our case series, we considered 60 as a cut-off value to evaluate psychologic readiness to return to sport six months after surgery [13]. Webster et al. followed up with 635 athletes 12 months after ACL reconstruction, and observed that greater limb symmetry while performing a single-legged hop for distance positively correlates with the ACL-RSI score [26]. Similarly, in the study by Aizawa et al. on athletes aiming to return to sport participation six months after surgery, LSI while performing jump tests affected the ACL-RSI score [25].

According to our findings, using individual changes with respect to baseline, patients with ACL-RSI score > 60 reported higher Lysholm and Tegner score and higher performance in the side-hop test six months after ACL surgery compared to patients with ACL-RSI score < 60. Sport-related performances such as vertical jump following ACL reconstruction are affected by muscular co-ordination recovery, leg power, and symmetry in isokinetic lower limb strength. Our study demonstrated that the ability to perform a side-hop test was significantly higher in the group with better psychological readiness. Similarly, patients with higher confidence tended towards higher PROMs compared to patients not meeting the ACL-RSI threshold of 60 points. This confirms our hypothesis that psychological readiness is strictly related to jumping ability. Interestingly, no difference with respect to subjective IKDC score, SJ, and CMJs were reported between the two groups considered. Further research is needed to investigate the most reliable predictors affecting ACL-RSI score at the time of return to sport.

Return to pre-injury activity level represents one of the most important issues in patients following ACL reconstruction. According to our results, average activity level did not statistically differ from pre-operative at follow-up, thus supporting the findings that most patients return to pre-injury activity level up to 12 months after surgery [28].

Future studies should build on current preliminary findings to help to evaluate return-to-sport readiness following ACL surgery, taking into account functional ability and psychological readiness when planning sport resumption. Long-term studies are needed to investigate prognostic factors which may allow for more appropriate decision-making strategies and give a reliable prediction on return-to-sport ability [29,30].

This study possesses limitations. The relatively small sample size may not have allowed for the detection of small differences between groups regarding some parameters. A trend for increased IKDC score in patients with ACL-RSI > 60 failed to demonstrate statistical significance. A greater number of patients could have enhanced the power of the results obtained. We acknowledge that jumping ability is influenced by many variables, and correlating jump height with neuromuscular restoration following ACL surgery is a further study limitation. The OptoGait device was chosen to instrumentally assess jump performance because it is simple, relatively inexpensive, and easily reproducible in the clinical setting allowing us to perform reliable measurements of functional ability. The male/female ratio of the patients recruited was biased towards the male gender, therefore our findings may not be generalizable to female athletes. Another limitation was the lack of a control group of healthy individuals. Finally, relying on subjective questionnaires could potentially bias the results. Long-term prospective follow-up studies with larger cohorts are required to corroborate these findings.

## 5. Conclusions

Patients with higher values of ACL-RSI scores showed better functional and clinical outcomes as well as improved performance six months after ACL reconstruction. Psychological readiness to return to sport reflects a better recovery of knee function following ACL surgery.

## Figures and Tables

**Table 1:** Patient demographics and anthropometric data.

	Overall	ACL-RSI > 60	ACL-RSI < 60	*p*-Value
No. of patients	31	20	11	
Gender Male Female	29 2	19 1	10 1	
Mean ACL-RSI score (SD)	68.3 (16.2)	78.2 (9.1)	50.2 (8.5)	
Mean age at surgery (SD) (yr)	34.6 (11.7)	34.7 (11.4)	34.4 (12.5)	0.9
Mean BMI (SD)	25.1 (3.2)	25.4 (3.4)	24.5 (2.7)	0.4

ACL-RSI: Anterior Cruciate Ligament Return to Sport after Injury; SD: standard deviation; BMI: Body Mass.

**Table 2:** Overall comparison between pre-operative and follow-up status.

	Pre-Operative	Follow-Up	*p*-Value
Lysholm score (mean, SD)	68.4 (15.6)	87.1 (11.2)	<0.001
Mean ACL-RSI score (SD)	51.9 (13.0)	77.1 (14.6)	<0.001
Mean age at surgery (SD) (yr)	46.2 (23.2)	68.3 (16.2)	<0.001
Tegner activity level (mean, SD)	4.2 (2.4)	5.1 (1.8)	0.161

SD: standard deviation; IKDC: International Knee Documentation Committee; ACL-RSI: Anterior Cruciate Ligament Return to Sport after Injury.

## Data Availability

Raw data will be provided on request.
